# Supplementary material for: Lipase-Catalyzed Production of Sorbitol Laurate in a “2-in-1” Deep Eutectic System: Factors Affecting the Synthesis and Scalability
Source: Molecules. 2021 May 7;26(9):2759. doi: 10.3390/molecules26092759 (PMC8124474; doi:10.3390/molecules26092759)
Supplement: Supplementary file 1 [file molecules-26-02759-s001.zip › molecules-1211424-supplementary/Supplementary/Supplementary Figure S8.pdf]

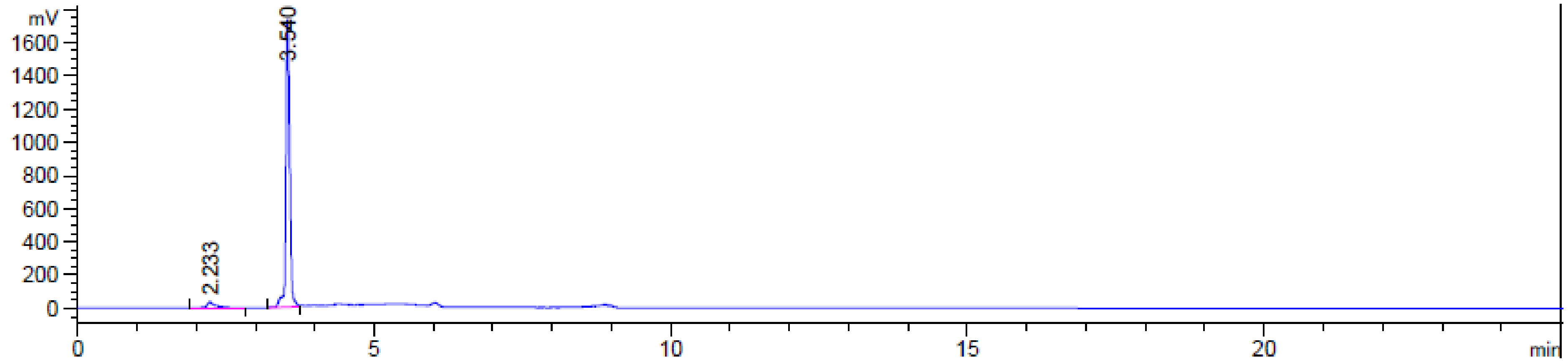

Supplementary Figure S8 Chromatogram for the purity assessment of the resulting sorbitol-6-O-laurate from the STR production using liquid-liquid extraction for DSP.
