# Supplementary figures and images for: Lipase-Catalyzed Production of Sorbitol Laurate in a “2-in-1” Deep Eutectic System: Factors Affecting the Synthesis and Scalability
Source: Molecules. 2021 May 7;26(9):2759. doi: 10.3390/molecules26092759 (PMC8124474; doi:10.3390/molecules26092759)

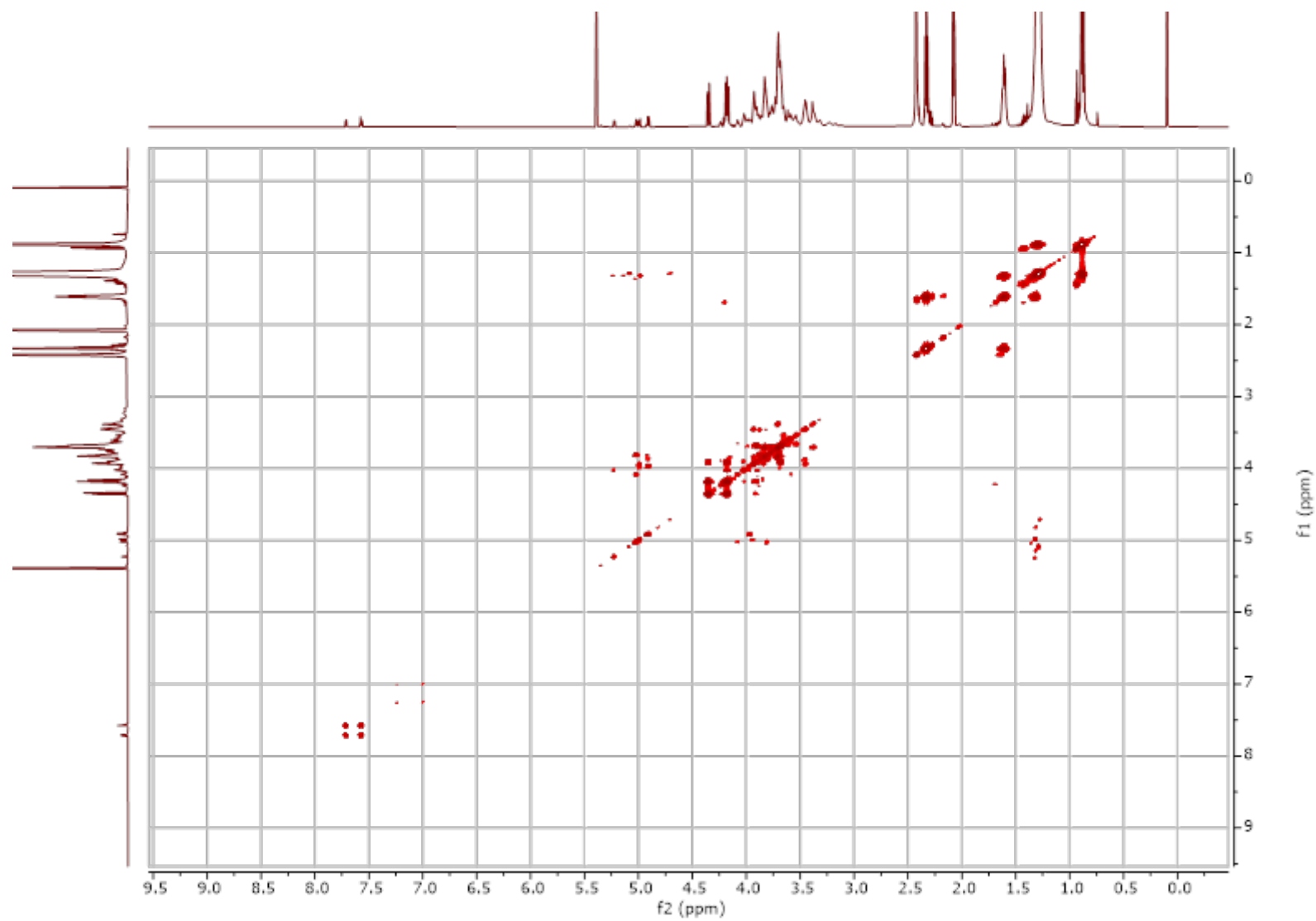

Supplementary Figure S2 2D NMR experiment:  $^1\text{H}$ - $^1\text{H}$  COSY of SL.

Supplement: Supplementary file 1 [file molecules-26-02759-s001.zip › molecules-1211424-supplementary/Supplementary/Supplementary Figure S2.pdf]

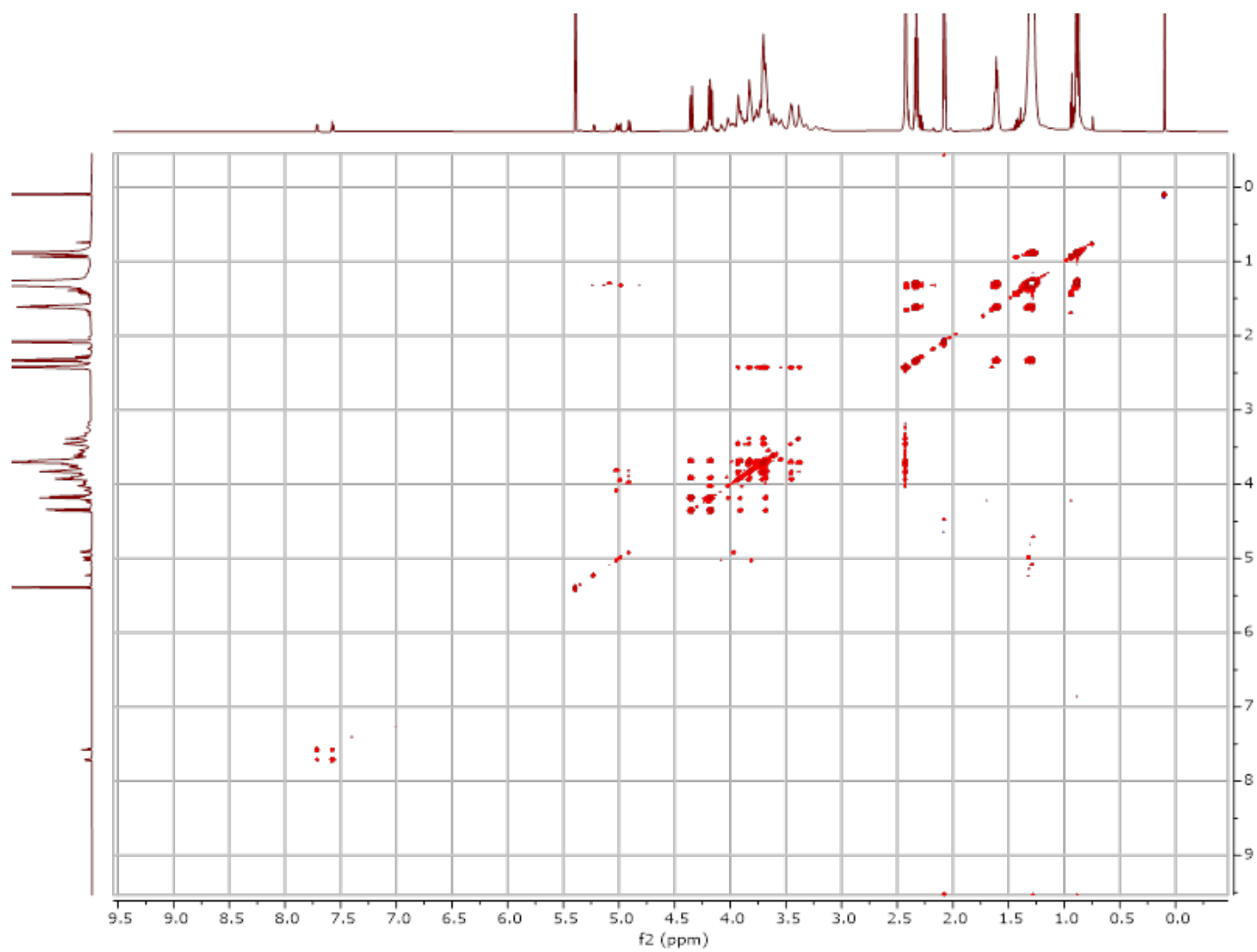

Supplementary Figure S3 2D-NMR experiment:  $^1\text{H}$ - $^1\text{H}$  TOCSY of SL.

Supplement: Supplementary file 1 [file molecules-26-02759-s001.zip › molecules-1211424-supplementary/Supplementary/Supplementary Figure S3.pdf]

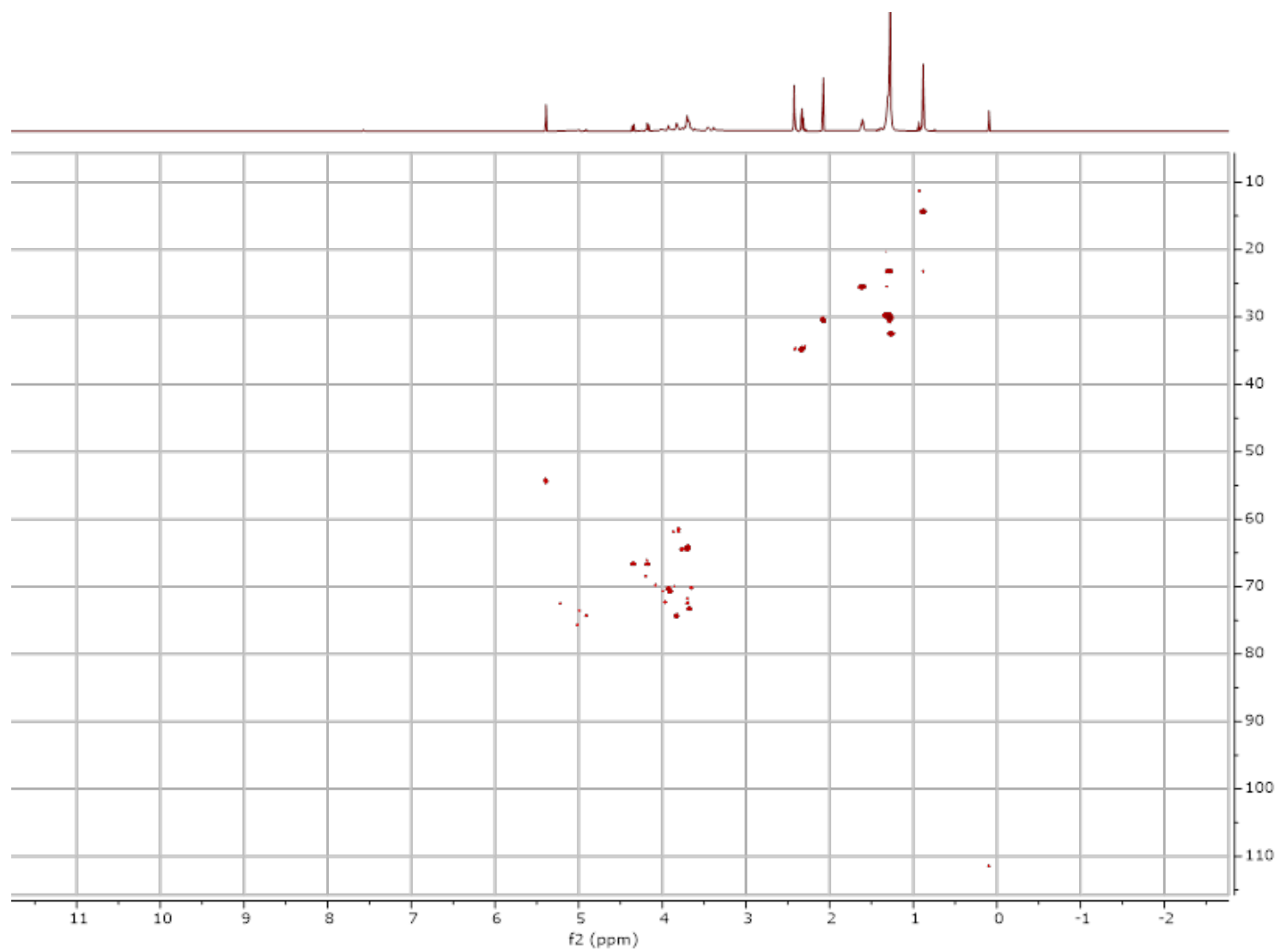

Supplementary Figure S4 2D-NMR experiment:  $^1\text{H}$ - $^{13}\text{C}$  HSQC of SL.

Supplement: Supplementary file 1 [file molecules-26-02759-s001.zip › molecules-1211424-supplementary/Supplementary/Supplementary Figure S4.pdf]

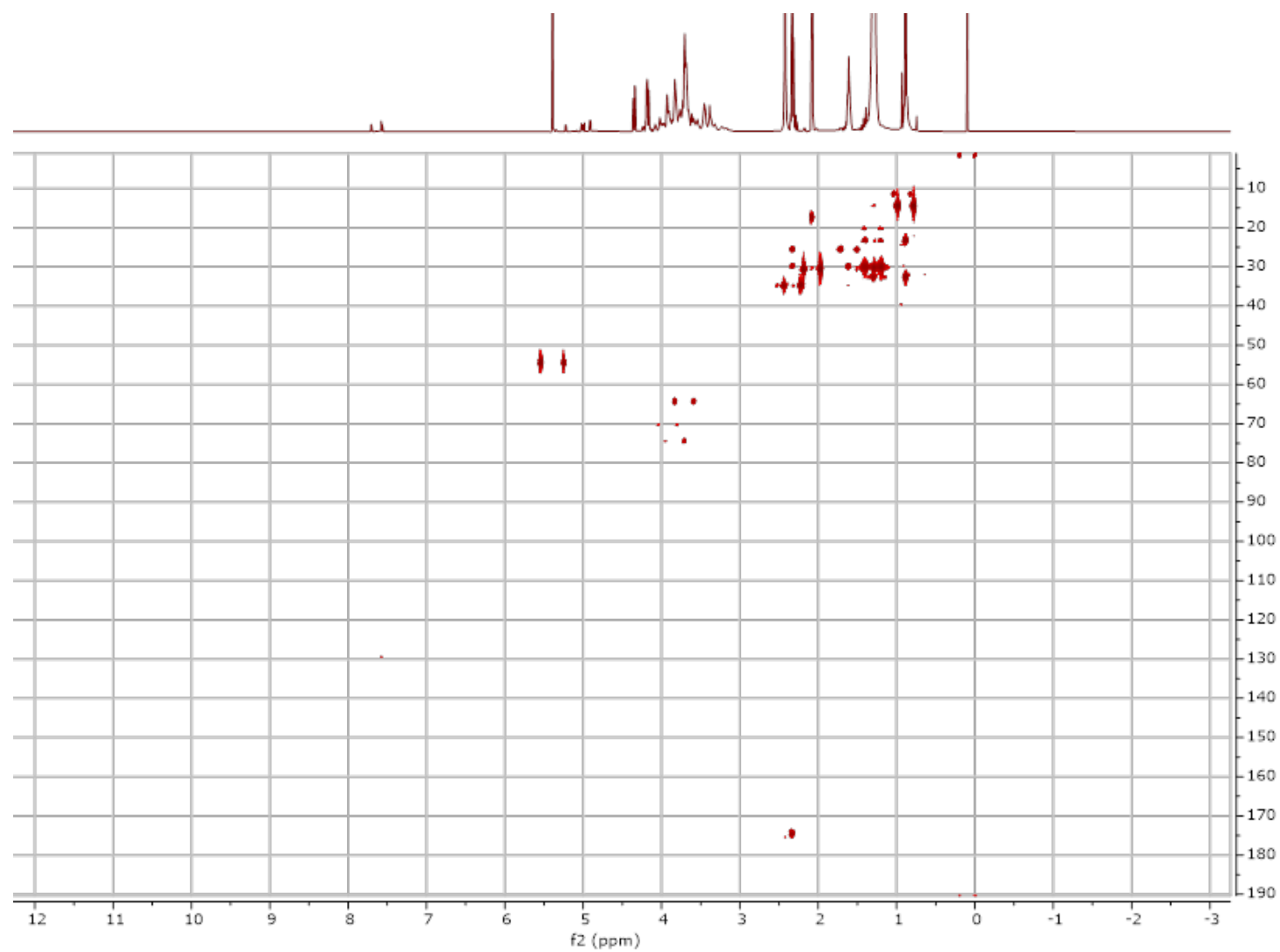

Supplementary Figure S5 2D-NMR experiment:  $^1\text{H}$ - $^{13}\text{C}$  HMBC of SL.

Supplement: Supplementary file 1 [file molecules-26-02759-s001.zip › molecules-1211424-supplementary/Supplementary/Supplementary Figure S5.pdf]
