# Supplementary material for: Lipase-Catalyzed Production of Sorbitol Laurate in a “2-in-1” Deep Eutectic System: Factors Affecting the Synthesis and Scalability
Source: Molecules. 2021 May 7;26(9):2759. doi: 10.3390/molecules26092759 (PMC8124474; doi:10.3390/molecules26092759)
Supplement: Supplementary file 1 [file Supplementary Figure S7.pdf › molecules-1211424-supplementary/Supplementary/Supplementary Figure S7.pdf]

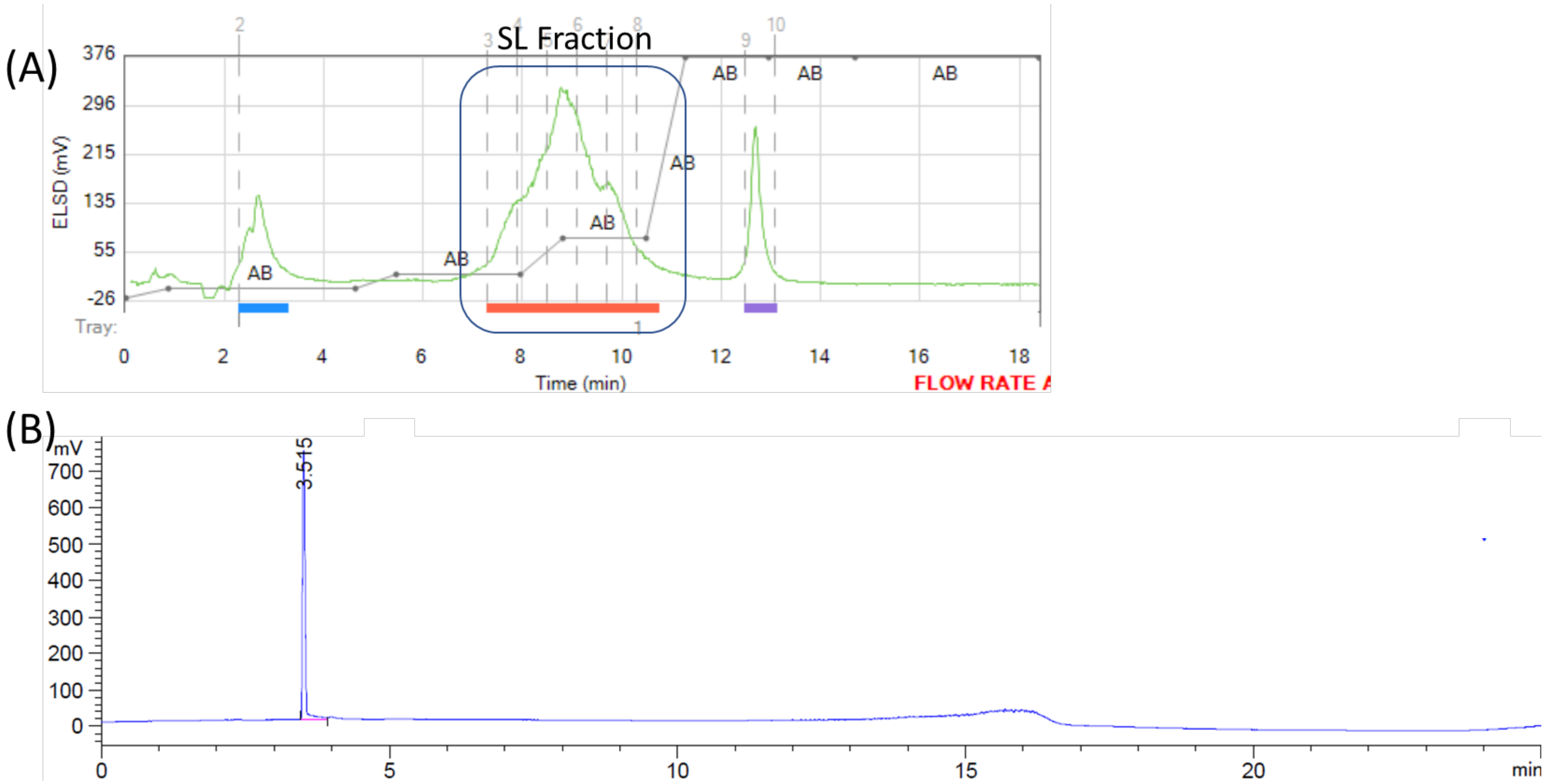

Supplementary Figure S7 (A) Chromatogram of the flash-chromatography purification of sorbitol-6-O-laurate. (B) Chromatogram for the purity assessment of the resulting sorbitol-6-O-laurate standard.
